# A New Ethical Challenge for Institutional Review Boards (IRBs)/Ethics Committees (ECs) in the Assessment of Pediatric Clinical Trials

**DOI:** 10.3390/children2020198

**Published:** 2015-05-28

**Authors:** Klaus Rose, Hans Kummer

**Affiliations:** 1 klausrose Consulting, Pediatric Drug Development & More, Aeussere Baselstrasse 308, 4125 Riehen, Switzerland; 2 University of Basel, Im Kirsgarten 57, 4106 Therwil, Basel-Landschaft, Switzerland; E-Mail: hmkummer@bluewin.com

**Keywords:** Better Medicines for Children, pediatric drug development, Ethics Committee (EC), Institutional Review Board (IRB), ethics, ethical clinical trials, unethical clinical trials, therapeutic orphans, therapeutic hostages, ghost studies

## Abstract

Both the US and EU have introduced pediatric pharmaceutical legislation to facilitate clinical trials in children and development of better medicines for children. The first concerns were published in 2014 that the European Medicines Agency (EMA)’s Pediatric Committee (PDCO) may be over-enthusiastic and has compelled questionable pediatric clinical trials from pharmaceutical companies. Numerous clinical trials are mandated in rare conditions for which not enough patients exist for even one trial. Furthermore, where these trials are mandated in adolescent patients, the legal age limit of the 18th birthday is confused with a medical age limit and can result in separate clinical trials in adolescent patients that neither make medical nor scientific sense nor will ever recruit enough patients for a meaningful outcome. To confirm our concerns we searched the registry clinicaltrials.gov and found examples for PDCO-triggered unethical trials. We conclude that such trials should not be accepted by institutional review boards (IRBs)/ethics committees (ECs) and that clinical trials resulting from negotiations with EMA’s PDCO need extra careful scrutiny by IRBs/ECs in order to prevent unethical studies and damage to pediatric research and unnecessary risks to pediatric patients.

## 1. Clinical Trials

Clinical trials are a key instrument to compare therapeutic interventions. To a relevant degree they have replaced experts’ opinion on what works, what does not work, and what works better. The methodology of clinical trials has evolved during the 20th century on the background of several, partially contradictory factors:

- Scientific curiosity *vs.* the rights and interests of the patients/healthy volunteers involved in clinical research
- Abuse of power, including war crimes of German and Japanese doctors in the second world war [1,2,3], criminal negligence of patients’ interests by the US public health service [4], by academic researchers, and acceptance of unethical trials by well-reputed journals [5].
- A clinical trial can provide evidence whether a given treatment is working or not. This will have a deep financial impact on a sponsoring company. Reporting a positive outcome of a new treatment can also have a significant career impact for a leading clinical investigator [6,7]. There are temptations for both to misrepresent, omit, twist or even falsify data.
- Randomized double-blind regulatory clinical trials have today a high weight in regulatory authorities’ decisions to approve new drugs. Clinicians will then prescribe them. Individual experience and learning is to some degree superseded by a complex system that includes approval of medicines by regulatory authorities and scientific agreement through literature, treatment guidelines, consensus papers, and more.
- To summarize, clinical trials play a key role in advancing medical science. However, clinical trials can also put patients into situations that can deeply affect their health and well-being, and key decisions are not immediately made by the treating physicians only. A thorough balance is required to allow on one side, progress in science, clinical understanding and therapeutic interventions, and on the other side, prevent damage to the individual patient, which in consequence, will damage the societal acceptance of clinical trials.

The backbone of the social acceptability of clinical trials has been formulated in several declarations and reports, including the original World Medical Association (WMA) declaration of Helsinki [8], in its respective current version, at present the one from the 64th WMA General Assembly, Fortaleza, Brazil, October 2013 [9]; the Belmont report that promulgated the key elements of respect for persons, justice, and benevolence [10]; the rules of good clinical practice (GCP) [11]; and more.

Drug development is complex [12], expensive [13], and controversial [14,15,16]. Nevertheless, few will doubt that there is progress in health care, which results in increased life expectancy in modern countries, and few will doubt that the availability of modern drugs and medical devices have a key role in this. To put it pointedly, without clinical trials, progress in modern health care would not be possible.

## 2. Clinical Trials in Children

Clinical trials in children have essentially the same features as in adults: they must follow the rules of GCP, including the key elements of the Belmont report and the Declaration of Helsinki. In addition, they must also deal with the different legal position of children in society, see also *Ethical Considerations for Clinical Trials on Medicinal Products Conducted with the Paediatric Population* [17]. These include the tenet that responsibility for health care decision lies with the parents. Furthermore, children have a different body and mind than adults. Both body and mind are in development.

An adult clinical trial protocol cannot be adapted to children by simply replacing the word ‘patient’ with ‘child’ or ‘pediatric patient’. This happened in the years after the first US pediatric legislation, when adult project teams in pharmaceutical companies were tasked with pediatric clinical trials in addition to their adult clinical trials workload. There were protocols which asked babies to sign informed consents, or where large amounts of blood were supposed to be taken from babies. This has changed; several books on clinical trials in children have been published in recent years [18,19,20,21].

There is broad agreement that healthy children should not participate in clinical trials. Measurement of ADME in children is always done in a clinical setting; e.g., the first test of a new antibiotic in children will be done where a traditional antibiotic is given, plus the new one, of which additional measurements on serum concentration and other parameters of absorption, distribution, metabolism, and excretion (ADME) are performed. Of course, in such a setting the study medication will start with a very low dose and will cautiously be escalated to doses assumed to be fully therapeutic.

## 3. Ethics Committees/IRBs

Ethics committees (ECs)/institutional review boards (IRBs) were introduced when it became apparent that the public trust in the integrity of academic and clinical institutions was not sufficient to protect patients. Before a clinical trial can begin today, it must be approved by the responsible EC/IRB. This gives the EC/IRB an enormous power. They are now a well-established institution to protect patients in clinical trials. They are local, which has advantages and disadvantages. Both drug development and medical science have become international and global, but health care will always remain local and hands-on. International clinical trials remind local clinical experts that there is a world outside of the territory where their opinion in medical questions is rarely challenged.

In a clinical trial, therapeutic key decisions are not made by the chief medical doctor alone. After initial diagnosis and checking inclusion and exclusion criteria, the respective treatment per patient is defined by the study protocol. This allows comparison of different treatments after study completion. In the EU, a new clinical trials regulation is currently in discussion that will hopefully provide binding procedural structures for all EU member states [22,23,24]. For local, monocentric clinical trials, this will not imply major changes. However, for multicenter international clinical trials, this should facilitate clinical trials in the EU.

## 4. Modern Drugs and Children

Never in the history of mankind has child health seen a higher place in public attention and clinical health care than today in developed western countries. Children with cancer have a high chance to survive—although still at a horrible price for their quality of life and that of their entire family. Vaccinations, antibiotics and antiviral substances have largely eradicated the horror of wards filled with children in iron lungs and of multiple early child deaths due to infectious diseases. The base for this progress was multiple and complex, including better nutrition in general and availability of nutrition for newborns where the mother did not have mother milk; availability of industrialized clothing; modern medicines, washing machines, and many more.

Modern drug labels originated from US legislation introduced in 1962 (Kefauver-Harris amendment to the Federal Food, Drug, and Cosmetic Act) [25]. Comparable legislation followed in Canada, European countries and Japan. Since then, labels reflect what has been proven in clinical trials and other measures in the laboratory or in animals. Before 1962, labels had only to describe the content of the package. From 1962 on, drug manufacturers introduced pediatric disclaimers that emphasized that the respective medicine had not been tested in children. In 1968, Henry Shirkey coined the term of children as ‘therapeutic orphans’, thus criticizing that children were not in the main focus of drug development [26,27,28]. In these days, companies often developed a drug in adults first, and maybe later in children. In the meantime, physicians prescribed these medicines off-label. After decades of discussion between scientists in academia, regulatory authorities and pharmaceutical industry, a first US pharmaceutical pediatric legislation in 1997 offered a patent extension in exchange for a pediatric clinical trials program negotiated with the FDA (FDAMA 1997) [29,30]. In 2003, legislation was introduced which gave the FDA the right to mandate pediatric clinical trials (PREA) [31]. Since 2012, both laws are in force without the need of regular re-authorization [32,33].

Ten years later, the EU introduced a comparable legislation [32,33,34,35,36]. It introduced a second procedural layer on top of the existing drug approval process. The EMA ‘paediatric committee’ (PDCO) was created. Before a company can submit a marketing authorization application (MAA), it must file a ‘paediatric investigation plan (PIP) and have it approved by the PDCO—unless the respective disease does not exist in children. For this there is an EMA list of ‘class waivers’, *i.e.*, diseases that are declared not to exist in children. The rest of the approval procedure is unchanged and the PDCO is not involved. Without an approved PIP, the EMA will not process a new MAA. While in the US rare diseases and vaccines are exempt from mandatory pediatric requirements, they need a PIP in the EU.

## 5. Reflections on Pharmaceutical Pediatric Legislation

In contrast to the clinical catastrophes in 1937 and 1962 and the ensuing revisions of the US pharmaceutical legislation [25], the US pediatric pharmaceutical legislation of 1997 with its re-authorizations through 2012 [32] was not introduced following a catastrophe. It was the result of a slow building up of academic pressure in the professional bodies of clinical pharmacology, pediatric clinical pharmacology, regulatory authorities, pharmaceutical industry, pediatric clinicians and others. It was the reaction to an increasing number of available new drugs where a lack of focus on child health was observed. It also reflected frustration in the daily life of clinical pharmacologists and pediatricians who had to explain every day to parents that this or that drug was not approved for use in children. High academic hopes accompanied pharmaceutical pediatric legislation [37,38,39,40,41].

However, many drugs have been developed specifically for children, including vaccines and conditions that exist in children only, e.g., lack of lung surfactant in preterm newborns or lack of growth hormone in children. For some time, the span of pharmaceutical development had focused on adult mass diseases, e.g., hypertension, dyslipidemia or gastric ulcer, where drugs were indeed developed for adults first and children later. Today, with new incentives offered for the treatment of rare diseases, we see many more new drugs developed for rare diseases in adults and children, e.g., treatment of Pompe’s disease, cystic fibrosis, enzyme deficiencies, and neurodegenerative diseases.

One of the greatest achievements of pediatric medicine in the 20th century was pediatric oncology. The road to this progress was not the merit of regulatory authorities. The pediatric oncologists tested adult anticancer drugs, mostly cytotoxic agents, in new doses and combination to children. They all were prescribed off-label. The merit of the regulatory authorities was to register them in adults. Many ‘classical’ chemotherapy agents used then are to this day still not registered in children. They are used off-label, have saved thousands of lives, and continue to do so [42,43,44,45,46,47].

There are many publications that justify the need for a pediatric pharmaceutical legislation with the percentage of drugs used off-label in children [18,19,20,21,40]. It is time to re-calibrate this debate. Off-label use has at least two sides. Used by pharmaceutical companies to expand sales into disease areas that have not been properly investigated, it is illegal and potentially damaging to patients. Off-label use can be recommended in disease areas or populations where there is strong scientific evidence of benefit—or the last hope in a life-or-death-situation.

IRBs/ECs should not be an obstacle against life-saving trials. Almost all children with cancer undergo treatment in the framework of clinical trials. In the late 20th century, the survival rate of, e.g., pediatric acute lymphatic leukemia (ALL) increased with each decade of the diagnosis by roughly 10%. The survival is today up to 90%. The last 10%, however, will not be achieved by further increasing the dose or by new combinations of toxic agents. Today’s challenge in pediatric oncology is the last 10%, as well as the quality of life of those that have to go through several cycles of chemotherapy, radiation and surgery.

Modern labels and the mandate to prove claims for safety and efficacy were key in the development of modern drugs. Without them there would not be a distinction between efficacious drugs and quack medicines. By its nature, pediatric pharmaceutical legislation cannot lead to breakthrough innovations needed for diseases that so far have defied modern treatment. These are malignancies in children, rare metabolic, genetic and other diseases that are increasingly detected by modern diagnostics. Pediatric legislation is an add-on to drug development, not more and not less. It needs to be handled by regulatory authorities in a balanced way.

## 6. Ethics Committees/IRBs and Clinical Trials in Children

Ethics committees face several specific challenges in regards to pediatric clinical trials. They have to protect children against various activities. These include academic clinical trials like the ones listed by Beecher 1966 [5]. IRBs/ECs also have to look critically at industry-sponsored trials that pretend to answer medical questions but essentially have a marketing purpose. If such a trial could harm patients, it should not be accepted. If the patient information makes false promises, the IRB/EC should force the applicant to revise it.

## 7. Ambitions of the European Medicines Agency (EMA) and its Paediatric Committee (PDCO)

The EU pediatric regulation came into force in 2007 [33,34]. Companies must negotiate a PIP with the EMA’s PDCO. Whenever a company wants to register a new drug, change the formulation, way of administration or indication of a registered medicine as long as there is any patent protection, it needs a PDCO-approved PIP [33,34,35,36]. In the US, biologics, vaccines and orphan drugs are exempt from mandatory pediatric requirements. Furthermore, the FDA will not try to force the company to develop its respective drug in different pediatric indication than the adult one.

The different EU approach reflects a belief that more pressure will help more. A negative opinion will block the registration of a new medicine. For the pharmaceutical industry, this is a deadly threat. And for every single patient with a disease that so far is untreatable, the blockage of a promising new treatment can result in a death sentence.

The EMA and the PDCO are full of good intentions and very enthusiastic. By the end of 2012, more than 1000 PIPs had been submitted, more than 600 were accepted, and many pediatric clinical trials were initiated by complying pharmaceutical companies. In contrast to the EMA's five-years-report, which praises itself as having marched from success to success, the EU Commission is more cautious in its 5-years-report: no significant impact on pediatric clinical practice yet, but give us more time [48,49].

Is every EMA/PDCO-imposed pediatric clinical trial in the pediatric patients’ best interests? In 2008 the EMA decided that there are enough adolescent patients with malignant melanoma to ask for clinical trials in this population and revoked the class waiver for melanoma [43,50,51,52]. However, the EMA calculations are wrong. The absolute frequency of patients with melanoma stated in the EMA decision is correct, as it quotes US statistics whose integrity is beyond any reasonable doubt. In these statistics adolescents are defined as 15–19 years old. But in EU law 18 and 19 year old patients are already adult. Furthermore, 75% of melanomas in young patients are in first world countries detected early and the melanoma is removed surgically at a stage where it is not yet metastasized. In other words: the EMA decision on the removal of the class waiver invokes a correct number of patients, but does not take into consideration that only about one tenth of these patients have a metastasized disease that makes them eligible for adolescent melanoma clinical trials that examine systemic treatment. The details of these miscalculations have been published in two peer-reviewed scientific papers and one editorial [43,50,51]. Melanoma is an adult disease that in rare cases can affect adolescent patients. These patients should have the right to participate in pivotal clinical melanoma trials. But to impose separate clinical trials for this population based on a legal age limit - the PDCO’s ‘territory’ is defined by patients from birth to the end of the 17th year of life - may legally be justifiable; medically it is not.

So far, five melanoma PIP decisions have been published on the EMA website that must recruit juvenile melanoma patients that need systemic treatment [53,54,55,56,57]. There are not enough patients in the EU and US for even one regulatory clinical efficacy trial in adolescent patients with metastasized malignant melanoma. Furthermore, such a trial would be unethical, because it defines trial participation on a legal age limit with disregard of the medical need. Melanoma in adolescents is very rare. Isolated cases should have the right to participate in an adult clinical trial. To enforce separate clinical trials is medically wrong and scientifically unethical. The terms ‘ghost study’ for this type of trial, and ‘therapeutic hostages’ for the few unfortunate patients that are recruited into such a trial were coined in 2014, alluding to Shirkey’s term of children as ‘therapeutic orphans’ [43,50,51]. Ghost studies are initiated at high costs by pharmaceutical companies to avoid EMA/PDCO’s negative verdicts and compliance checks, trying desperately to recruit very rare, almost non-existing patients. After a few years, the EMA PDCO will then be humbly re-approached with the request to modify the PIP. At the end, the trial is terminated without any meaningful results.

To verify our concerns, we looked for clinical studies in adolescent patients with melanoma in the clinical trials registry of the US National Library of Medicine (NLM), worldwide the largest clinical trials database. It is accessible online under www.clinicaltrials.gov. As of 4 March 2015, the search terms ‘adolescent melanoma’ shows seven studies, for details see Table 1.

**Table 1:** Clinical Trials in Adolescent Melanoma on www.clinicaltrials.gov.

Study ID	Study Title	Sponsor	Intervention	Patient Age	Centers
NCT00743496	A Phase I Trial Of The Humanized Anti-GD2 Antibody In Children And Adolescents With Neuroblastoma, Osteosarcoma, Ewing Sarcoma and Melanoma	St. Jude Children’s Research Hospital	Anti-GD2 antibody	Up to 21 years	1 US Center
NCT01696045	Phase 2 Study of Ipilimumab in Children and Adolescents (12 to < 18 Years) With Previously Treated or Untreated, Unresectable Stage III or Stage lV Malignant Melanoma	Bristol-Myers Squibb	Ipilimumab	12–17 years	32 centers USA, UK, Australia, Mexico, Belgium, Denmark, France, Germany
NCT01677741	A Study to Determine Safety, Tolerability and Pharmacokinetics of Oral Dabrafenib In Children and Adolescent Subjects [including melanoma patients]	Glaxo-Smith-Kline	Dabrafenib	12 months–17 years	17 Centers USA, Canada, France, Spain, UK
NCT01508013	An Appearance-Based Intervention to Reduce Teen Skin Cancer Risk (iSTART)	East Tennessee State University	Behavioral: Appearance-Focused Website Intervention	13–18 years	1 US Center
NCT01709435	Cabozantinib in Treating Younger Patients With Recurrent or Refractory Solid Tumors [including melanoma]	National Cancer Institute	Cabozantinib	2–18 years	23 Centers in USA & Canada
NCT00931931	HSV1716 in Patients With Non-Central Nervous System (Non-CNS) Solid Tumors [including melanoma]	Nationwide Children’s Hospital	Biological: HSV1716	7–30 years	2 US Centers
NCT02147080	A Tailored Internet Intervention to Reduce Skin Cancer Risk Behaviors Among Young Adults (UV4me)	Fox Chase Cancer Center	Behavioral: UV4me/Skin Cancer Foundation website	18–25 years	1 US Center

Two studies aim at preventing behavior by adolescents that carries the risk of later melanoma, including indoor tanning, sunless tanning creams, *etc.* Of the remaining five studies, three are run and sponsored by public US institutions, and two are sponsored by pharmaceutical companies. These are the investigated agents:

- The antibody used in trial NCT00743496 is directed against CD2, which is expressed on the surface of most refractory neuroblastoma, osteosarcoma, Ewing sarcoma, and melanoma tumor cells, which are considered difficult to treat and cure.
- Ipilimumab is FDA approved since March 2011 for late-stage melanoma that has spread or cannot be removed by surgery. In Canada, it is approved for treatment of unresectable or metastatic melanoma in patients who have failed or do not tolerate other systemic therapy for advanced disease. It is EMA-approved for second line treatment of metastatic melanoma since November 2012.
- Dabrafenib is FDA-approved as a single agent for BRAF V600E mutation-positive advanced melanoma since May 2013.
- Cabozantinib is FDA-approved since November 2012 for medullary thyroid cancer and is currently undergoing clinical trials for multiple other solid cancer types, including melanoma.
- HSV1716 is an oncolytic virus, a mutant herpes simplex virus (HSV) type I, deleted in the RL1 gene which encodes the protein ICP34.5, a specific determinant of virulence. Mutants lacking the RL1 gene are capable of replication in actively dividing cells but not in terminally differentiated cells—a phenotype exploited to selectively kill tumor cells. Replication of HSV1716 in human glioblastoma *in situ* has been demonstrated. Following a single administration of HSV1716 by direct injection into active recurrent tumor or brain adjacent to tumor, some patients have lived longer than might have been expected. This study seeks to evaluate the safety of a single injection of HSV1716 in the treatment of extracranial solid tumors in adolescents and young adults.

Both ipilimumab and dabrafenib are already FDA-approved in patients 18 years and older. There is no medical sense in recruiting adolescent patient into an extra trial. The age limit of 18 years is a *legal*, not a medical age limit. There is no medical rationale to treat an 18 year old patient with a licensed medicine but to refuse treatment to an adolescent patient who is half a year younger. In the dabrafenib trial, it could be argued that ADME data and maximally tolerated dose (MTD) data are necessary in the very young patients. But then a totally different trial would need to be designed. The currently running PIP-triggered pediatric melanoma trials have no meaningful questions to answer and should therefore be regarded as unethical. Ethics committees/IRBs should have refused these two trials. These two trials are triggered by EMA PDCO PIP decisions [53,55]. They are not trials organized with the aim of successfully treating patients or at least aiming at prolonging their lives or improving their quality of life. Instead, they are imposed on two companies that, in order to comply with the EMA PIP decisions, now recruit patients worldwide. For pharmaceutical companies, this is a catch-22 situation. Whatever they do, they do something wrong. If they open a trial they will be criticized because these trials make no medical sense. If they do not, they will be challenged by EMA/PDCO. Interestingly, there are three more melanoma PIPs for which trials have not even started—otherwise they would be registered in www.clinicaltrials.gov.

The two trials run and sponsored by pharmaceutical companies compete with the three other academic clinical trials that aim at other ways to treat adolescent melanoma. It can be anybody’s guess which chance the academic trials will have in this bizarre hunt for rare adolescent patients.

ECs/IRBs should reject ghost studies that abuse patients as therapeutic hostages. There is danger of ghost studies in PIPs that concern a disease that is rare in children. We would like to encourage ECs/IRBs to have a specifically critical view at clinical trials resulting from a PIP negotiation and have been accepted by a company under duress: threatened with non-registration of their new medicine due to the lack of an agreed PIP. Such trials need to be looked at with double scrutiny, specifically as far as they concern rare pediatric forms of diseases that are frequent in adults or are rare diseases in general. This double scrutiny should also be applied by ECs/IRBs if a clinical trial is to be performed in adolescents only or where adolescents are included into a clinical trial. Skin, joints, liver, heart and many other organs of adolescents are medically mature, *i.e.*, adult—in contrast to adolescents’ brain and reproductive organs, which are still in development. Separate trials in adolescents that investigate organ systems that are already essentially mature do not answer meaningful medical question, and should be rejected.

## 8. Conclusions

Drug development is a complex multi-billion dollar business performed by competing companies. We have seen enormous progress in science and health care over the past 100 years and an increased life expectancy in most countries, even more so in the developed world. Children should benefit from this progress. ECs/IRBs were created when blind trust in the researchers’ integrity was obviously not sufficient to prevent abuse of vulnerable patients. This paper highlights a new challenge, *i.e.*, overzealous insistence to conduct pediatric clinical trials, which comes from an unexpected source. New challenges do not always originate from expected sources. IRBs/ECs will in the coming years have an even higher responsibility to make sure that children are not abused as therapeutic hostages, just for the feeling that ‘something is done’. If the EU wants to contribute seriously to better child health, it could increase the respective research and clinical care budgets and/or offer better incentives to pharmaceutical industry to develop better medicines for children and adults. Ethics committees remain a key barrier in modern society against unethical abuse in unnecessary clinical trials in children and adolescents.
